# Standardization of acoustic measures for normal voice patterns

**DOI:** 10.1016/S1808-8694(15)31023-5

**Published:** 2015-10-19

**Authors:** Ana Clara Naufel de Felippe, Maria Helena Marotti Martelletti Grillo, Thaís Helena Grechi

**Affiliations:** a MS. PhD - FFCLRP-USP. Voice Specialist - CFFa. Professor of Speech and Hearing Therapy-Unaerp.; b MS in Education - Voice Specialist - CFFa. Professor of Speech and Hearing Therapy-Unaerp.; c Specialist in Orofacial Movement- Professor of Speech and Hearing Therapy-Unaerp Especialista em Motricidade. Linked to the Course of Speech and Hearing Therapy - Universidade de Ribeirão Preto (UNAERP).

**Keywords:** acoustic measures, normal voice, standardization

## Abstract

Studies have established that normative data is necessary for acoustic analysis. The aim of the present study is to standardize fundamental frequency measures (fo), jitter, shimmer and harmonic-noise ratio (HNR) for young adults with normal voice.

**Method:**

20 males and 20 females, between 20 and 45 years, without signs and symptoms of vocal problems; CSL-4300 Kay-Elemetrics; vowels /a/ and /é/.

**Results:**

for females, vowels /a/ and /é/ had average measures of: fo 205.82 Hz and 206.56 Hz; jitter of 0.62% and 0.59%; shimmer of 0.22 dB and 0.19 dB; PHR of 10.9 dB and 11.04 dB, respectively. For males, vowel /a/ and /é/ had average measures of: fo 119.84 Hz and 118.92 Hz; jitter of 0.49% and 0.5%; shimmer of 0.22 dB and 0.21 dB; HNR 9.56 dB and 9.63 dB, respectively. Both fo and NHR female measures were significantly higher than their male counterparts.

**Conclusion:**

our results differ from the literature; therefore, it is important to standardize the program in use.

## INTRODUCTION

Acoustic analysis is one of the components of computerized voice labs, and it is useful to supplement voice assessment^1^, ^2^ and to assess speech^3^, ^4^, ^5^.

Many are the acoustic parameters evaluated in this analysis, and the most commonly used for voice assessment are: fundamental frequency, jitter, shimmer and the harmony-noise ratio.

The fundamental frequency is an important parameter in both the functional and anatomical larynx assessment^6^, and it is determined by the number of cycles produced by the vocal folds per second. Such measure is the result of the iteraction among vocal fold length, mass and tension during speech. Among acoustic parameters, fundamental frequency has proven to be the most uniform of them when we consider different acoustic analysis systems, and the one less sensitive to voice recording characteristics^7^, ^8^, ^9^.

Frequency and cycle-to-cycle amplitude variation measures, jitter and shimmer, respectively, in the production of sustained vowels have proved to be useful in the description of normal and dysphonic speakers’ vocal characteristics, being respectively related to hoarseness and roughness^6^, ^10^, ^11^, ^12^, ^13^. Fundamental frequency, jitter and shimmer seem to also suffer the influence of smoking - fundamental frequency is significantly lower and both jitter and shimmer are higher when we compare smokers to non-smokers^14^.

The harmony-noise ratio characterizes the relationship between the two components of the acoustic wave of a sustained vowel: the periodic component, vocal fold regular sign and the additional noise coming from the vocal folds and the vocal tract^15^, ^16^.

This ratio is also significantly different between genders, being higher for females^17^, and it is also influenced by age, being lower for the elderly (from 70 to 90 years), when compared to a group of young (from 21 to 34 years) and middle age women(from 40 to 63 years)^16^, but it is not a sensitive parameter to differentiate the dysphonic from the normal voice.^13^.

In Brazil, acoustic analysis has been more intensely used in the last decade. Casmerides and Costa^18^ carried out a study with 32 speech therapists who worked with voice, all of them were professors of Speech Therapy, in order to characterize this group of users, and found that 47% were interested in solving their clinical needs. This was the reason why they used acoustic analysis programs as a complementary tool in their practice. As a general opinion, they attempted to obtain less subjective and more quantitative data. Another result from such study was that, despite the fact that the users seemed to be worried about the quality of recorded samples, standardization did not occur among the users of the same type of lab, nor among the users of different lab types.

According to Titze^19^, standardization educates; simplifies; saves time, money and effort, and assures certification.

Knowing that speech and voice computerized analysis programs use different modes to calculate acoustic parameters, some studies attempt to standardize data for their equipment^6^, ^10^, ^17^, ^20^, ^21^ and others have compared their main acoustic measures among the different analysis programs, trying to know whether or not there is an agreement among them^7^, ^22^, ^23^.

Karnell et al.^22^, compared the fundamental frequency, jitter and shimmer among 3 programs and found a measure agreement for the fundamental frequency, but not for jitter and shimmer.

Morris e Brown^7^ compared 6 different acoustic analysis systems in order to assess their reliability levels and the agreement among them in determining the fundamental frequency. Their results pointed towards high reliability in each one of the systems when they repeated the assessment of the same signal; however, the agreement among signals varied, being high for the fundamental frequency in men when they uttered a sustained vowel and also in oral reading for women, but low agreement for oral reading in men and sustained vowel for women. The authors also found that the CSL program proved to be the most accurate system to measure the fundamental frequency for the sustained vowel /a/; notwithstanding, it has the highest level of standard deviation, specially for vowel /a/.

Aiming at determining and comparing fundamental frequency values, jitter and shimmer of female individuals, through 4 acoustic wave analysis methods, Spinelli and Behlau^23^ assessed 24 subjects without signs and prior history of vocal alterations when uttering the sustained vowel /a/. Results have shown that the fundamental frequency values were similar only between the Soundscope software and stroboscopy, which in turn were lower than the values found by the Vocal-2 software and higher than those found by the Dr. Speech software. Values for jitter and shimmer determined by the Soundscope and Dr. Speech software were statistically different.

Since the literature shows that there are many variables which compete for the final result of a computerized acoustic analysis, it is necessary to normatize the specific data from the software we are utilizing.

Thus, the goal of the present study was to normatize fundamental frequency, jitter, shimmer and noise-harmony ratio (NHR) measures for the CSL 4300 software, from Kay Elemetrics, used in the Speech Therapy Clinic of Ribeirão Preto, so as to obtain comparison data for voice analysis.

## METHOD

This study was approved by the Ethics Committee for Research of the Ribeirão Preto University (protocol # 10/03). The subjects were informed about the goal, procedure and disclosure of its results. After agreeing, they signed an informed consent approved by the aforementioned committee and in agreement with Resolution # 196/96 Ministry of Health/ National Health Board/ National Committee of Research Ethics (MS/CNS/CNEP).

Forty young adults, 20 men and 20 women took part in this study. They all went to the University of Ribeirão Preto: they were employees, students, or they were accompanying patients who were going to the Speech Therapy Clinic. Minimum age was of 20 years, since puberty brings about voice alterations stemming from the voice change. Maximum age was of 45 years, because of possible voice changes caused by the very aging of the vocal apparatus as of this age. Age is a relevant variable in vocal assessment^16^.

Other selection criteria for the subjects included not having any signs and symptoms of voice change and not smoke^14^. The procedure used to assess the selection criteria was a questionnaire answered by the participant prior to sample collection (Attachment 1).

Besides not presenting voice alterations signs and symptoms (checked by the questionnaire), the participant's voice was also assessed by the same two speech therapists (paper authors) and only data from the individuals considered with normal voice became part of the present study.

Data collection was carried out in a sound treated room, using the acoustic analysis software CSL-4300 from Kay-Elemetrics, at the Speech Therapy Clinic of the Ribeirão Preto University. The microphone used was a Shure SM 48 dynamic, and it was kept at a fixed distance of 5 cm in front of the subject's mouth. We used the sustained vowels /a/ and /é/, in a comfortable and habitual way, after deep inhaling. The sustained vowel is preferred over regular speech in vocal acoustic assessment^24^. When the sample differed from the regular subject's voice, a new sample was collected. Vocal intensity was controlled by monitoring the software's Vu meter.

In order to analyze the samples, we used the time of 3 seconds, and both the beginning and end of the vowel uttering were discarded. We also discarded the samples in which the authors found altered voice quality.

These vowels were analyzed as to their acoustic parameters: fundamental frequency (Hz), jitter (%), shimmer (dB) and noise-harmony ratio (NHR) (dB). Each one of these parameters was analyzed as to gender and vowel.

The statistical data analysis was carried out through SAS^25^ GLM procedure, considering the variance analysis mathematical model for random outlining, in split plots (split plot)^26^, using the following expression:

Where yijk = value observed regarding the in gender, from the jn subject, in the kn vowel; m = fixed factor, estimated by the general average; Ii = effect of the in gender (i = female and male); eij = random error corresponding to the plots, supposedly homocedastic, independent and normally distributed; Vk = effect of the kn vowel (k = /a/ and /e/); (SV)ik = effect of the interaction between the in gender with the kn vowel; eijk = random error, corresponding to the subplots, supposedly homocedastic, independent and normally distributed. The minimum level of significance used was of 5% (p£0.05).

## RESULTS

Table 1 depicts the likelihood descriptive levels of the F test for the values assessed.

**Table 1:** Likelihood descriptive levels of the F test, variation coefficients and measures for the fundamental frequency (fo), Jitter, Shimmer and NHR.

Causes for variation	GL	Variables (Pr>F)			
		Fo (Hz)	Jitter (%)	Shimmer (dB)	NHR (dB)
Gender 1	1	<,0001	0,0865	0,5259	0,0360
Vowel 1	1	0,8954	0,6323	0,1106	0,8157
Gender*Vowel 1	1	0,2776	0,3134	0,4500	0,9443
Variation Coefficient (%)	–	2,0339	18,066	20,822	19,118
Average	–	162,78	0,5555	0,2160	10,286

GL = Degrees of Freedom.

**Graph 1:**
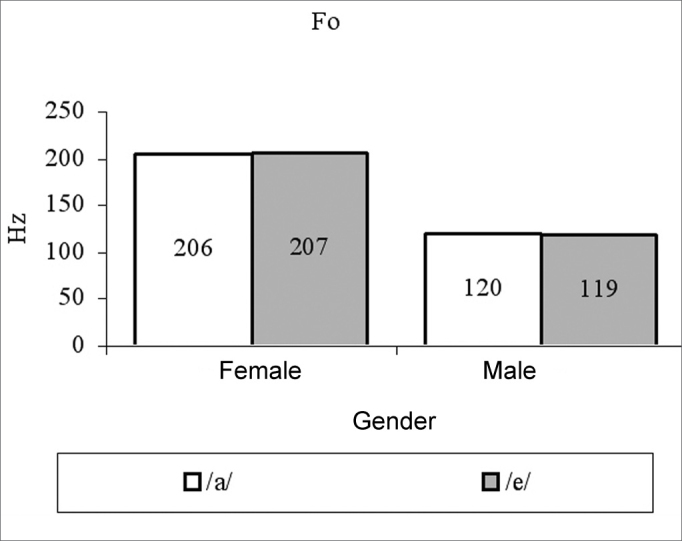
Fundamental frequency averages (f0) in function of gender and vowel.

**Graph 2:**
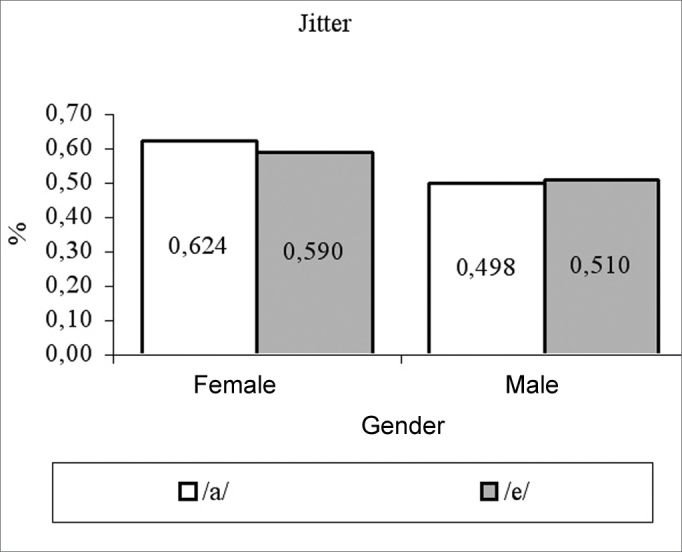
Jitter measures in function of gender and vowel.

**Graph 3:**
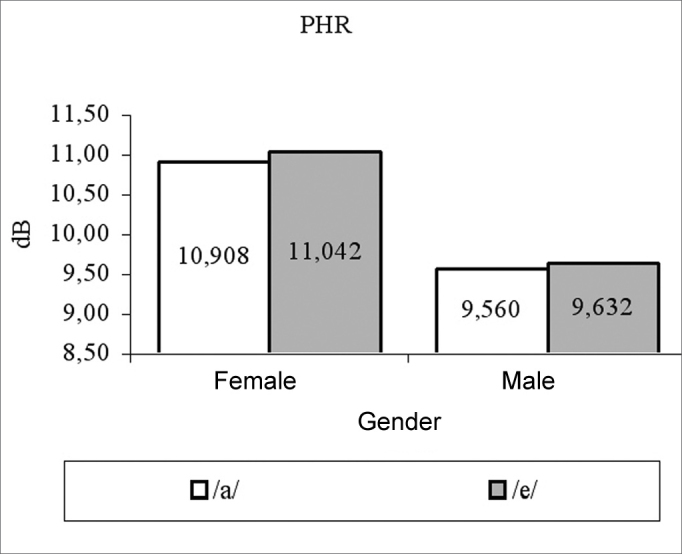
Averages of the noise-harmony ratio related to gender and vowel.

**Graph 4:**
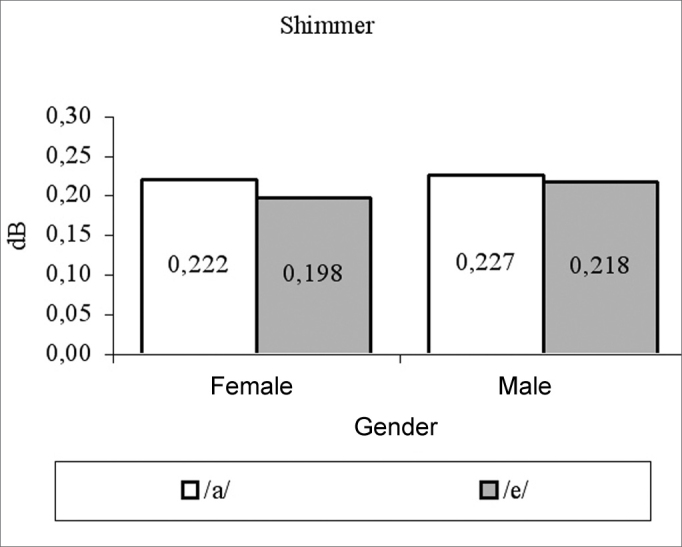
Shimmer measures in function of gender and vowel.

We can see in Table 1 that the vowel factor and its interaction with gender was not significant (p>0.05) in all the cases, there has been a significant effect for the gender factor only on variables fo (p<0.0001) and NHR (p=0.0360), and the female averages were higher than their male counterparts for these variables (Graphs 1, 4). For jitter and shimmer, although female averages were higher than the male averages, they did not differ among themselves (p=0.0865) (Graphs 2, 3).

## DISCUSSION

The fundamental frequency average found in the present study for vowel /a/, in men (120Hz) was lower than the ones found by Horii^10^ - 125Hz, by Araújo et al.^20^ - 127.61Hz, by Morente et al.^13^ - 139.72Hz and higher than the one found by Behlau and Tosi^21^ - 113.01Hz. The average of the same parameter for women, 206Hz, was lower than the one found by Araújo et al.^20^ - 215.42Hz and the one found by Morente et al.^13^ - 267.33Hz; however, it was very similar to the ones found by Ferrand^16^, which were 209.68Hz for young women and 204.49Hz for middle aged women.

The significant difference in the fundamental frequency average values in function of gender, found in the present study was expected, since it is influenced by the length of the vocal folds, which is longer in males. This difference has been often pointed out in the literature^20^, ^21^.

The jitter average regarding vowel /a/, for men was 0.498%, lower than the one found by Horii^6^, ^10^, which were 0.61% and 0.66%, respectively; however, it was higher than the average found by Tajada^14^ - 0.23% and by Araújo et al.^20^ - 0.37%. As to the average jitter regarding vowel /a/, for females, our result (0.62%) was lower than the one found by Araújo et al.^20^ - 0.85%, but similar to the one found by Ferrand^16^ - 0.69%.

The shimmer average for men, producing the vowel /a/, was 0.23dB, and such value was lower than the one found by Horii^6^, which was 0.47 dB and the one found by Araújo et al.^20^, which was 2.37dB, but greater than the one found by Horii^10^, which was 0.132dB. Average shimmer for females producing the vowel /a/, in the present study was 0.22dB. This value was much lower than the one found by Araújo et al.^20^, which was 2.52dB.

The jitter averages in function of gender were not significantly different, although the female gender presented a value that was below the one presented by males. Behlau and Tosi^21^ found a similar result and they also found it difficult to launch the hypothesis about which would be the reasons for this better sound control shown by women. These authors raised the possibility that it could happen because females use their voices more frequently, and this could be seen as training.

This lack of difference in the gender-related average jitter values corroborate other studies^6^, ^13^, ^21^; however it disagrees from another one that found the average jitter value of 0.37% for men and 0.85% for women^20^. As to shimmer, there was no difference as far as gender was concerned in the present study, and such data was also found in other papers^20^, ^21^.

The average of the noise-harmony ratio for men and women in the present study, when producing the vowel /a/, was respectively 9.56dB and 10.98dB, values above the ones found by Rodrigues et al.^17^, which were 8.63dB and 10.17dB and to the ones from Ferrand^16^, who found the value of 7.82 dB for young women. Just like it happened in the present study, Rodrigues et al.^17^, found a significant difference between genders, in other words, that women presented values significantly higher for the noise-harmony ratio in comparison to men. It may be that this is related to the fact that men use fluid voice more frequently, which is characterized by lesser glottal closure, and this favors voice production with less harmonics and or greater amount of glottal noise. In normal voices, basal recording is associated to a greater noise level^17^ and since such recording is more frequent in males, this would justify the result found.

In general, our results are similar only to those found by Ferrand^16^, maybe it is because they used the CSL model 4300 software, from Kay Elemetrics, as we did in the present study. The other studies mentioned used other acoustic analysis programs, such as Dr. Speech Science^13^, ^14^, ^23^, Soundscope^17^, ^23^, Matlab^12^, Vocal II^23^, Kay Elemetrics 5500 DSP^22^ and a software developed by the Federal University of São Carlos^20^.

Knowing of the possible value difference in acoustic parameters between different analysis software, some authors studied the issue^7^, ^22^, ^23^. For fundamental frequency, one study found agreement among the software^22^, another found it only in sustained vowels for men, but not for women^7^ and a third one found an agreement in the fundamental frequency values between the software Soundscope and Stroboscopy, but not between Dr. Speech and the Vocal II^23^. When we compare shimmer and jitter values from the different software, we have noticed a certain variability^22^, ^23^, and this makes it unfeasible to use the standards of a certain software in another.

Besides these differences among the different software, the recording criteria, the microphone, the way by which the programs calculate the parameters are factors that generate variations in the acoustic parameters. We should also consider the cultural variations that affect speech and voice, for example a higher or lower pattern in voice production.

The difference between our results and those from other authors confirm the need to normatize each software to be used.

We lack the references to discuss the values found for the vowel /é/, since the authors approaches did not use this vowel, but we also did not find significant differences among the vowels /é/ e /a/ in the present study.

## CONCLUSION

The average values of normality found in the present study for male voices producing the vowel /a/ were fo = 120Hz, jitter = 0.498%, shimmer = 0.23dB and NHR = 9.56dB and producing the vowel /é/ were fo = 119Hz, jitter = 0.591%, shimmer = 0.218dB and NHR = 9.632dB. The average values found for females voices, producing the vowel /a/ were f0 = 206Hz, jitter = 0.62%, shimmer = 0.22dB and NHR = 10.98dB and for vowel /é/ were f0 = 207Hz, jitter = 0.590%, shimmer = 0.198dB and NHR = 11.04dB.

The differences in the programming of the various acoustic analysis systems, as well as the use of recording criteria and computers, microphones and other devices make each one of these systems a single one, thus, precluding a single normatization. Therefore, users should base themselves on their own normatization.
